# Children with premature pubarche: is an alterated neonatal 17-Ohp screening test a predictive factor?

**DOI:** 10.1186/s13052-018-0444-6

**Published:** 2018-01-16

**Authors:** Paolo Cavarzere, Margherita Mauro, Monica Vincenzi, Silvana Lauriola, Francesca Teofoli, Rossella Gaudino, Diego Alberto Ramaroli, Rocco Micciolo, Marta Camilot, Franco Antoniazzi

**Affiliations:** 1 Pediatric Division, Department of Pediatrics, University Hospital of Verona, Verona, Italy; 2 Department of Pediatrics, Neonatal Intensive Care Unit, University Hospital of Verona, Verona, Italy; 3 Department of Psychology and Cognitive Sciences, University of Trento, Trento, Italy; 4 Regional Center for the diagnosis and treatment of children and adolescents rare skeletal disorders, Pediatric Clinic, Department of Surgical Sciences, Dentistry, Gynecology and Pediatrics, University of Verona, Verona, Italy

**Keywords:** Premature pubarche, 17-Ohp, Newborn screening, Congenital adrenal hyperplasia

## Abstract

**Background:**

Neonatal screening for 21 hydroxylase deficiency is designed to detect classical form of congenital adrenal hyperplasia (CAH). It is still unclear whether newborns who result false positives at neonatal screening might later develop signs of androgen excess. The aim of this study is to verify whether a slightly elevated 17-OHP at newborn screening is a predictive factor for premature pubarche.

**Methods:**

We evaluated all infants born between 2001 and 2014 with premature pubarche. In case of increased bone age, they were submitted to functional tests to find out the cause of their symptoms. Their 17-OHP values at newborn screening for CAH were reconsidered.

**Results:**

We identified 330 patients (269 females, 61 males) with premature pubarche. All these children had a normal 17-OHP at newborn screening with the exception of a child, born preterm and not affected by CAH.

**Conclusions:**

An elevated 17-OHP at newborn screening is not a predictive factor for premature pubarche. A likely cause of increased 17-OHP level at screening is an immaturity of adrenal gland or a neonatal stress. Therefore a strict follow up of these neonates during childhood is not necessary.

## Background

Since 2001 a neonatal screening program for congenital adrenal hyperplasia (CAH), based on 17-hydroxyprogesterone (17-OHP) assay on filter paper-dried blood spots, has been instituted in North-Eastern Italy. It aims to obtain an early identification of the newborns affected by classic form of 21 hydroxylase deficiency (21-OHD), and consequently to prevent both potential life-threatening shock due to salt waste, especially in male newborns, and incorrect sex assignment in affected girls with inappropriate virilization [1–4]. Nevertheless, the real sustainability of this neonatal screening remains debated. Some studies have in fact underlined the high rate of recall that the neonatal screening determines, especially in preterm infants, and pointed out the psychological and financial costs resulting in these recalls, which entail a visit to a pediatrician and serum confirmatory tests [5, 6]. In order to prevent these drawbacks, we have previously suggested to avoid any further investigations in newborns who result positive at CAH neonatal screening and do not present classic form of CAH, when serum 17-OHP levels decrease over time after birth [7]. However, screening procedure does not allow to identify babies with non-classical 21-OHD, since basal serum 17-OHP levels are often in the normal range in patients with this form of disease [1, 8–11]. Consequently, it is recommended to perform other more specific confirmatory assessments, namely functional tests, if further symptoms, such as premature pubarche, acne, hirsutism, appear [1, 3, 7]. In fact, it is still unclear whether a newborn with a slightly elevated 17OHP levels at neonatal screening may later develop premature pubarche or other symptoms of androgen excess. Furthermore, a precocious identification of premature pubarche is useful since it may reduce related complications like obesity and insulin resistance [12, 13].

Hence, the aim of this study is to verify whether an elevated 17-OHP at newborn screening is a predictive factor for premature pubarche.

## Methods

We enrolled in this study all infants born between 2001 and 2014 that were later referred to the Pediatric Endocrinology Division of Verona Hospital, Italy, for premature pubarche defined as development of pubic hair before the age of 8 years in girls or 9 years in boys.

All patients were evaluated in order to determine their weight, height, BMI, and pubertal development using Tanner and Whitehouse method [14]. For boys, testicular volume was assessed using a Prader’s orchidometer. All children underwent radiography to determine bone age, evaluated with Greulich and Pyle method [15]. In children with advanced bone age, blood tests were performed to measure serum 17-OHP, cortisol, testosterone, ACTH, DHEAS, Δ4-androstenedione. Moreover, gestational age (GA), birth weight (BW), birth length (BL) and result of 17-OHP screening were reconsidered.

ACTH stimulation test was performed using soluble Synacthen 250 mg intravenously for all children with increased bone age (ratio of bone age to chronological age greater than 1). Serum levels of 17-OHP and cortisol were evaluated at baseline and after 60 min. Data were analyzed using Maria New nomogram [16].

GnRH-analogous (GnRH-a) stimulation test was performed in patients with associated signs of precocious puberty (premature thelarche for girls or testicular volume > 4 mL for boys), administering 0.1 mg of GnRH-a (Triptorelin) subcutaneously after overnight fasting. Serum levels of FSH, LH, estradiol and testosterone were measured at baseline and 4 h after the injection.

Serum 17-OHP was assayed by radioimmunoassay (DSL-5000 Active 17α-OH Progesterone Coated-Tube Radioimmunoassay, Diasorin Spa Italia). Assay sensitivity was 0.01 ng/dL, whereas intra- and inter-assay coefficients of variation were both 9.0%.

Serum total testosterone, ACTH, cortisol, Δ4-androstenedione, DHEAS, estradiol, FSH and LH were measured by a solid-phase, competitive chemiluminescent enzyme immunoassay (Immulite 2000, Siemens Healthcare Diagnostic, Deerfield, IL, USA). Analytical sensitivity was 0.05 mIU/mL (0.5 nmol/L) for total testosterone, 1.1 pmol/L for ACTH, 0.2 μg/dL for cortisol, 1 nmol/L for Δ4-androstenedione, 0.3 μg/dL for DHEAS, 0.1 mIU/mL for FSH, 0.05 mIU/mL for LH and 15 pg/mL for estradiol.

Intra-assay and inter-assay coefficients of variation were 11.7 and 13.0% respectively for testosterone, 9.5 and 10.0% respectively for ACTH, 7.4 and 9.4% respectively for cortisol, 9.3 and 12% respectively for Δ4-androstenedione, 4.1 and 6.3% respectively for DHEAS, 2.9 and 4.1% respectively for FSH, 3.6% and 6.7 respectively for LH, 11.7 and 13% respectively for estradiol.

Neonatal screening for CAH was performed using dried blood spots on filter paper. The blood sampling was collected between 48 and 72 h of life. 17-OHP was determined by immunofluorometric assay (Kit DELFIA Neonatal 17-hydroxyprogesterone Kit; Perkin Elmer, Wallac Oy, Turku, Finland).

The study was conducted in compliance with the terms of the Helsinki II Declaration. In Italy, this type of retrospective study do not require Institutional Review Board/Institutional Ethics Committee approval in order to publish the results. Written informed consent was obtained from the parents of each patient.

All statistical analyses were performed using software R. Comparisons between groups were performed using Student’s *t*-test or the Wilcoxon test, when appropriate. Data are expressed as numbers with frequency, median plus range, or mean ± SD, as appropriate. Statistical significance was considered when *p*-values were less than 0.05.

## Results

We identified 330 patients with premature pubarche, 269 females (81.5%) and 61 males (18.5%). Their mean age at diagnosis was 7.3 ± 1.8 years. The auxological data of our population are represented in Table 1. Twenty percent of them were premature with GA < 37 weeks and the 13.7% had low body weight (< 2500 g). Thelarche in association to pubarche was present in the 31% of girls, while only 3% of male patients had an enlargement of testicular volume. Hundred and seventy-two patients (51.5%) presented advanced bone age. In female patients bone age was 1.3 ± 1.0 years increased compared to anagraphic age, in boys the increase of bone age was of 1.4 ± 1.0 years. These 172 patients underwent blood exams as shown in Table 2.

**Table 1:** Neonatal and auxological data at diagnosis. Data are expressed as mean ± standard deviation and range (min-max) in the brackets

	Patients
GA (weeks)	38.5 ± 2.5 (25.0–42.0)
BW (g)	3059 ± 650 (787–5000)
BL (cm)	49.5 ± 2.8 (25.0–55.0)
Age at diagnosis (years)	7.3 ± 1.8 (0.4–10.3)
wheight SDS at diagnosis	3.1 ± 2.2 (−1.6–11.4)
height SDS at diagnosis	2.0 ± 1.2 (−1.7–5.4)
BMI SDS at diagnosis	2.1 ± 2.1 (−2.2–9.9)

*GA* Gestational Age, *BW* Birth Weight, *BL* Birth Length

**Table 2:** Laboratoristic exams. Data are expressed as mean ± standard deviation and range (min-max) in the brackets

	Patients
Cortisol basal (μg/dL)	11.3 ± 8.0 (1.6–63.0)
Cortisol peak (μg/dL)	26.4 ± 5.5 (1.0–42.9)
ACTH (pg/mL)	28.5 ± 42.2 (6.2–373.0)
17 OHP basal (ng/dL)	1.3 ± 2.4 (0.1–18)
17 OHP peak (ng/dL)	5.2 ± 11.6 (1.1–110.0)
Testosterone (ng/dL)	20.3 ± 6.8 (0.3–39.6)
Testosterone peak (ng/dL)	22.3 ± 14.7 (2.1–72.8)
Δ4-androstenedione (ng/mL)	0.6 ± 0.5 (0.3–3.0)
DHEAS (μg/dL)	74.6 ± 48.8 (0.4–291.0)

**Fig. 1:**
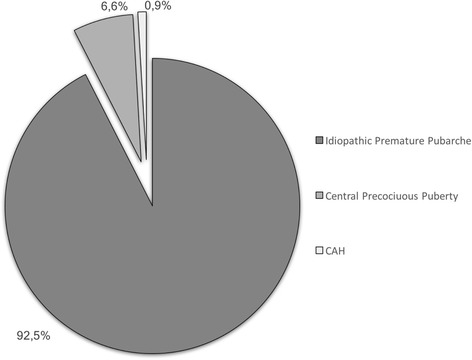
Etiology of premature pubarche in our cohort

Figure 1 represents the etiology of pubarche identified in our cohort on the basis of ACTH test and GnRH-a test results (data not shown). Moreover, 25 children (24 females and 1 male) presented levels of 17-OHP at the ACTH test compatible with a heterozygous condition for 21-OHD.

**Fig. 2:**
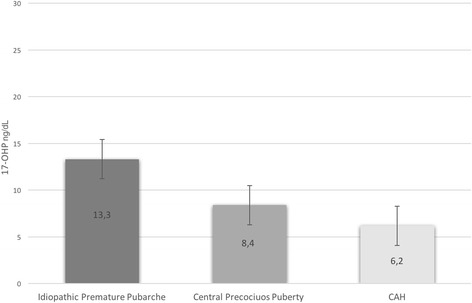
Mean 17 OHP levels at neonatal screening of patients with premature pubarche, depending on their etiology (*p > 0.05*)

Figure 2 represents the 17 OHP levels evaluated at neonatal screening of all patients with premature pubarche. All 3 patients with late onset CAH had normal 17-OHP screening values. Only one patient (born preterm at 28 weeks of GA and with BW of 1010 g) presented borderline values of 17-OHP at newborn screening; however, he was not affected by CAH but only by an idiopathic premature pubarche.

## Discussion

To our knowledge, this is the first study analyzing the relation between premature pubarche and the 17-OHP level at newborn screening for CAH.

Premature pubarche is not rare and usually appears as secondary condition of an early isolated maturation of adrenal gland [17–19]. In some patients, adrenal androgens may be moderately increased for chronological age, although in the normal range for their stage of pubic hair, in others androgen levels are normal, but some Authors have suggested an increased peripheral sensitivity to androgens [17]. Nevertheless, the diagnosis of premature pubarche is a diagnosis of exclusion. In fact, in the 6.6% of our patients, premature pubarche was associated with precocious puberty, since all these children presented not only premature pubarche, but also some initial pubertal signs. Moreover, late-onset CAH, other rare mild errors of steroidogenesis and virilizing adrenal or gonadal tumors must all be ruled out [17].

Within a diagnostic frame, it remains controversial which patients should be submitted to ACTH stimulation test. Some Authors recommend to submit patients to this test as the ratio between bone age and statural age exceeds 1, or whenever androgen levels are elevated, or, finally, in case of bone age advancement, cystic acne or other signs of systemic virilisation detection [17]. We decided to perform the ACTH test whenever the ratio of bone age to chronological age was greater than 1 and, on this basis, we identified 3 children affected by late-onset CAH (1.7%). None of our cohort presented virilising tumors.

The remaining children (92.5% of total cohort) resulted affected by idiopathic premature pubarche. Among them, the 63.5% presented elevated DHEAS levels. Although DHEAS is a weak androgen, it is a substrate for the synthesis of more potent androgens, and levels of DHEAS superior than 40–50 μg/dL are associated with the appearance of a pubarche [17, 19, 20]. In these patients premature pubarche may originate from a functional adrenal hyperandrogenism of unknown aetiology, perhaps due to hyperplasia of the zona reticularis of the adrenal gland. In the other 36.5% the appearance of the premature pubarche may be related to the above mentioned increased peripheral sensitivity to normal androgen levels.

The prevalence of CAH in our cohort of children with premature pubarche is comparable with previous data, which have estimated it to a range from 0 to 40% [9, 20–24]. Yet, after ACTH stimulation test, 25 children (14.5%) showed level of 17-OHP compatible with a heterozygous condition. It is well known that patients with premature pubarche, hirsutism and early puberty may present a 21-hydroxylase mild deficiency due to the heterozygous status [25]. As a matter of fact, a high percentage of subjects unaffected by CAH but presenting hyperandrogenic signs carries mutations of the CYP21A2 gene in heterozygous state [26–28]. Whereas some Authors report similar risk for mutation carriers and wild type subjects [29, 30], others evidenced an association between clinical signs of hyperandrogenism and the heterozygous condition, especially in subjects carrying the mutation V281 L [31, 32]. A dominant-negative effect of this mutant allele on the wild type has been described, reducing the enzyme activity with the consequence of a higher risk of symptoms due to androgen excess [31]. Although we did not submit our 25 children to genetic analysis, their 17-OHP levels compatible with heterozygous condition would confirm an association between heterozygous condition and the presence of premature pubarche. The real mechanism behind this association still remains unknown.

With the exception of one child born preterm, all patient with premature pubarche, including the 3 non classical CAH patients, presented 17-OHP levels in the reference range at newborn screening. The data presented in this manuscript show that an elevated 17-OHP at newborn screening is not a sign of increased risk of premature pubarche. As above mentioned, neonatal screening for CAH is not designed to detect non-classical form of the disease, since basal serum 17-OHP levels are often in the normal range in patients with this form of disease [1, 8–11]. Their identification might require a lower cut-off value, with a significant increase in false-positive recalls and substantial rise in the cost:benefit ratio [33]. But is it necessary to submit asymptomatic neonates with only slightly increased serum 17-OHP levels to complete exams in order to exclude middle forms of CAH, which in neonatal period may not require any treatment but only an adequate follow-up over time? In a previous manuscript, we concluded that no further investigations are necessary in newborns with a positive screening test if their serum 17-OHP levels decrease, and we suggested to make other confirmatory tests only later on, if further symptoms appear [7]. Other Authors have suggested an annual follow-up by paediatric endocrinologist in order to check growth rate and signs of CAH [1, 34, 35]. Our data permit not to recommend a periodic endocrinologist follow up for these newborns; further endocrinological investigations being necessary only if symptoms appear during childhood.

The major drawback of neonatal screening for 21-OHD are the high false-positive rate and the low positive predictive value, mainly among preterm, low BW and ill neonates [4, 36–38]. In particular, in France the efficiency of routine 21-OHD screening resulted very low in preterm neonates, therefore French Authors suggested to interrupt the screening in these newborns [6]. However, to reduce the false-positive rate, other screening programs have established 17-OHP cut-off levels in relation to GA [36, 39–41] or BW [42–44]. We also used a cut-off associated to GA [45]. The only child with idiopathic premature pubarche presenting elevated 17-OHP at newborn screening resulted, in fact, unaffected by CAH; however, he was born preterm (28 weeks of GA), and, as well known, increased 17-OHP levels are common in preterm and low BW infants [36, 37, 46]. Since the expression of the enzyme 11-β-hydroxylase physiologically appears with some delay [47], it is above all premature newborns with a GA of less than 31 weeks that have elevated screening 17-OHP levels without inborn errors of steroid biosynthesis [48]. In a previous study, we have hypothesized that a low 21-hydroxylase enzymatic function might cause the elevated 17-OHP level evidenced at screening and we have affirmed that this enzymatic immaturity might also be present in term babies [7]. Other factors that may contribute to increase the 17-OHP level at birth are neonatal jaundice or neonatal disease. In fact, hyperbilirubinaemia may contribute to higher 17-OHP values due to the effect of dehydration on blood concentration [49], and neonatal stress may increase ACTH and 17-OHP levels [41].

## Conclusions

In conclusion, we exclude that an elevated 17-OHP evidenced in babies unaffected by classical 21-OHD at newborn screening might be a sign of future premature pubarche. The main identified causes of increased 17-OHP levels at birth remain an immaturity of adrenal gland and/or a neonatal stress. Consequently, a strict follow up of these infants during childhood is not necessary.

## Abbreviations

17-OHP: 17-hydroxyprogesterone
21-OHD: 21 hydroxylase deficiency
BL: Birth length
BW: Birth weight
CAH: Congenital adrenal hyperplasia
GA: Gestational age
GnRH-a: GnRH-analogous
SD: Standard deviations
